# Molecular detection of *Mollicutes* agents in reproductive system of cattle: a three-year study in Rio Grande do Sul, southern Brazil (2022–2024)

**DOI:** 10.1007/s11250-026-04940-y

**Published:** 2026-03-05

**Authors:** Helena Castro Alves Wessely, Evelyn Kaus Dotto, Alice Sampaio Moraes da Costa, Fernanda Silveira Flôres Vogel, Juliana Felipetto Cargnelutti, Alfredo Quites Antoniazzi

**Affiliations:** 1 Centro de Ciências Rurais, Programa de Pós-Graduação de Medicina Veterinária, Universidade Federal de Santa Maria, Santa Maria, Rio Grande do Sul CEP 7105900 Brasil; 2 Centro de Ciências Rurais, Graduação em Medicina Veterinária, Universidade Federal de Santa Maria, Santa Maria, Rio Grande do Sul CEP 7105900 Brasil; 3 Centro de Ciências Rurais, Departamento de Medicina Veterinária Preventiva, Universidade Federal de Santa Maria, Santa Maria, Rio Grande do Sul CEP 7105900 Brasil; 4 Centro de Ciências da Saúde, Departamento de Fisiologia e Farmacologia, Universidade Federal de Santa Maria, Santa Maria, Rio Grande do Sul CEP 7105900 Brasil

**Keywords:** *Mollicutes*, *Mycoplasma* spp., *Ureaplasma diversum*, Cattle, Vulvovaginitis, PCR

## Abstract

Rio Grande do Sul state, in southern Brazil, is one of the country’s largest cattle producers but reports few cases of genital infections caused by *Mollicutes*, specially *Ureaplasma diversum* and *Mycoplasma bovis*. The clinical signs associated with these bacteria can be easily confused with those caused by bovine herpesvirus (*Varicellovirus bovinealpha1*), *Campylobacter fetus*, and *Tritrichomonas fetus*, which complicates diagnosis. This study aimed to assess the occurrence of *Mollicutes* in cattle from Rio Grande do Sul between April 2022 and November 2024. Vulvo-vaginal and preputial lavage samples submitted for routine diagnosis at a Bacteriology Laboratory, were tested using PCR. Samples were first tested for *U. diversum* and *M. bovis*. Those testing negative were further examined for *Mycoplasma bovigenitalium* and *Mycoplasma* spp., and if still negative, for the *Mollicutes* class to detect other species. In total, 136 samples from 16 beef cattle farms in central, southern, and western regions of the state were analyzed. These included herds with reproductive disorders (*n* = 11) and asymptomatic animals (*n* = 5). *U. diversum* was detected in 44 samples (32%) from nine herds, both with and without reproductive issues. *M. bovis* was identified in one sample (1%) from a herd with reproductive problems. *M. bovigenitalium* was found in 11 preputial samples (9%) from three affected herds. PCR also detected *Mycoplasma spp.* in 25 samples (18%) and *Mollicutes* class in 31 samples (23%). These results confirm the presence of *Mollicutes* in reprodutive system in both, symptomatic and asymptomatic animals, highlighting their circulation in herds from Southern Brazil.

## Highlights

Mollicutes infect cattle reproductive tracts in southern Brazil.

Asymptomatic cattle can silently spread Mollicutes.

Mollicutes infections mimic other reproductive pathogens, complicating diagnosis

## Introduction

Beef cattle farming represents a significant component of Brazil’s national agribusiness sector. In 2024, it contributed approximately 8.4% to the country’s gross domestic product, generating an estimated R$ 987.36 billion through the production of 10.2 million tons of beef (ABIEC, 2025). In the same year, the national cattle herd was estimated at 238,180,757 head, with the state of Rio Grande do Sul accounting for 11,530,056 animals, approximately 3.2% of the national total, according to the most recent data from the Brazilian Institute of Geography and Statistics (IBGE) (BRASIL, 2024; SECRETARIA DE PLANEJAMENTO, GOVERNANÇA E GESTÃO, 2024). Sector productivity, however, is influenced by a range of factors across the production chain, among which reproductive performance plays a pivotal role. Within this context, reproductive health issues such as infertility, embryonic loss, and abortion have a direct impact on both productivity and farm profitability (Antoniassi et al. 2007). Then, the routine molecular diagnostics and sanitary measures (herd health monitoring) could be readily implemented to better manage reproductive issues and reduce economic losses across the beef production chain.

Infectious and parasitic diseases are the primary causes of reproductive losses in cattle (Alfieri and Alfieri 2017). Among the primary pathogens associated with reproductive disorders worldwide are *Pestivirus bovis*, *Varicellovirus bovinealpha1*, *Brucella abortus*, *Leptospira interrogans*, *Campylobacter fetus*, and *Neospora caninum* (Tramuta et al. 2011; Derdour et al. 2017). However, less commonly investigated microorganisms can also induce infections and/or lesions in the reproductive tract of cows and bulls, compromising reproductive performance and leading to infertility, abortion, reduced pregnancy rates, and return to estrus (Alfieri and Alfieri 2017).

*Mollicutes* is a bacteria class characterized by fastidious growth in culture media, small size, highly compact genomes, and lack of a rigid cell wall, which confers resistance to numerous antimicrobials (Kuppeveld et al. 1993). Members of this class, such as *Mycoplasma bovis*, *Mycoplasma bovigenitalium*, and *U. diversum*, are associated with reproductive infections in cattle (Karl-Erik Johansson & Bertil Pettersson, 2002; Azevedo et al. 2017; Parker et al. 2018). These opportunistic pathogens colonize the mucosa of the vulva, vagina, and udder, causing severe granular vulvovaginitis, salpingitis, endometritis, mastitis, placentitis, and fetal alveolitis, potentially resulting in temporary infertility, spontaneous abortion, or weak calves’ birth (Doig et al. 1980). In bulls, infections are often asymptomatic but can occasionally cause balanoposthitis, epididymitis, and seminal vesiculitis (Junior et al. 2021). Transmission occurs through natural mating and artificial insemination with contaminated semen (Cardoso et al. 2000).

Genital infections caused by *Mollicutes* in cattle have been well-documented (Petit et al. 2008; Lysnyansky et al. 2009; Argue et al. 2013; Ghanem et al. 2013; Gaeti et al. 2014). In Brazil, laboratory detection remains limited, likely due to the fastidious nature of these bacteria and their specific culture media requirements (Kuppeveld et al. 1993). This limitation may contribute to underdiagnosis, leading to an apparent low prevalence in herds. Existing studies in Brazil have primarily focused on identification and genetic characterization (Gaeti et al. 2014; Azevedo et al. 2017; Carli et al. 2021) or diagnostic assays development (Voltarelli et al. 2018). In Rio Grande do Sul State (southern Brazil), one of the largest cattle-producing regions in the country, reports of genital infections caused by *Mollicutes* are scarce (Carli et al. 2021). As a result, current epidemiological situation data may not accurately reflect the prevalence of these bacteria, particularly in cows.

Given the above, this study aimed to detect and identify the occurrence and distribution of class *Mollicutes* bacteria in clinical samples collected from beef cattle farms in Rio Grande do Sul between April 2022 and November 2024.

## Material and methods

### Experimental design and clinical samples

A total of 136 vulvovaginal swabs and preputial lavage samples were collected from beef cattle across 16 farms in the central, southern, and western regions of Rio Grande do Sul between April 2022 and November 2024. These samples were submitted for routine diagnosis at the Bacteriology Laboratory of the Federal University of Santa Maria for microbiological diagnosis due to a history of reduced pregnancy rates, return to cyclicity, vesicular lesions on the vulvovaginal mucosa, or for routine herd health monitoring.

The samples were analyzed for molecular detection of *Mollicutes*. They were then stored in saline solution and frozen at -20 °C until further processing.

### DNA extraction

DNA extraction from vaginal swab samples was performed using the genomic DNA mini kit (PureLink, Invitrogen^™^, USA) following the manufacturer’s protocol. DNA from preputial lavage samples was extracted using a reagent (DNAzol, Invitrogen^™^, USA). Extracted DNA was stored at -20 °C until PCR analyses.

### Primers and PCR

Before the *Mollicutes* polymerase chain reaction (PCR), all samples tested negative for *Varicellovirus bovinealpha1*, *Campylobacter fetus*, and *Tritrichomonas foetus* (Hum et al. 1997; Felleisen et al. 1998; Mayer et al. 2006). The primers used for *Mollicutes* detection are presented in Table 1.

**Fig. 1:**
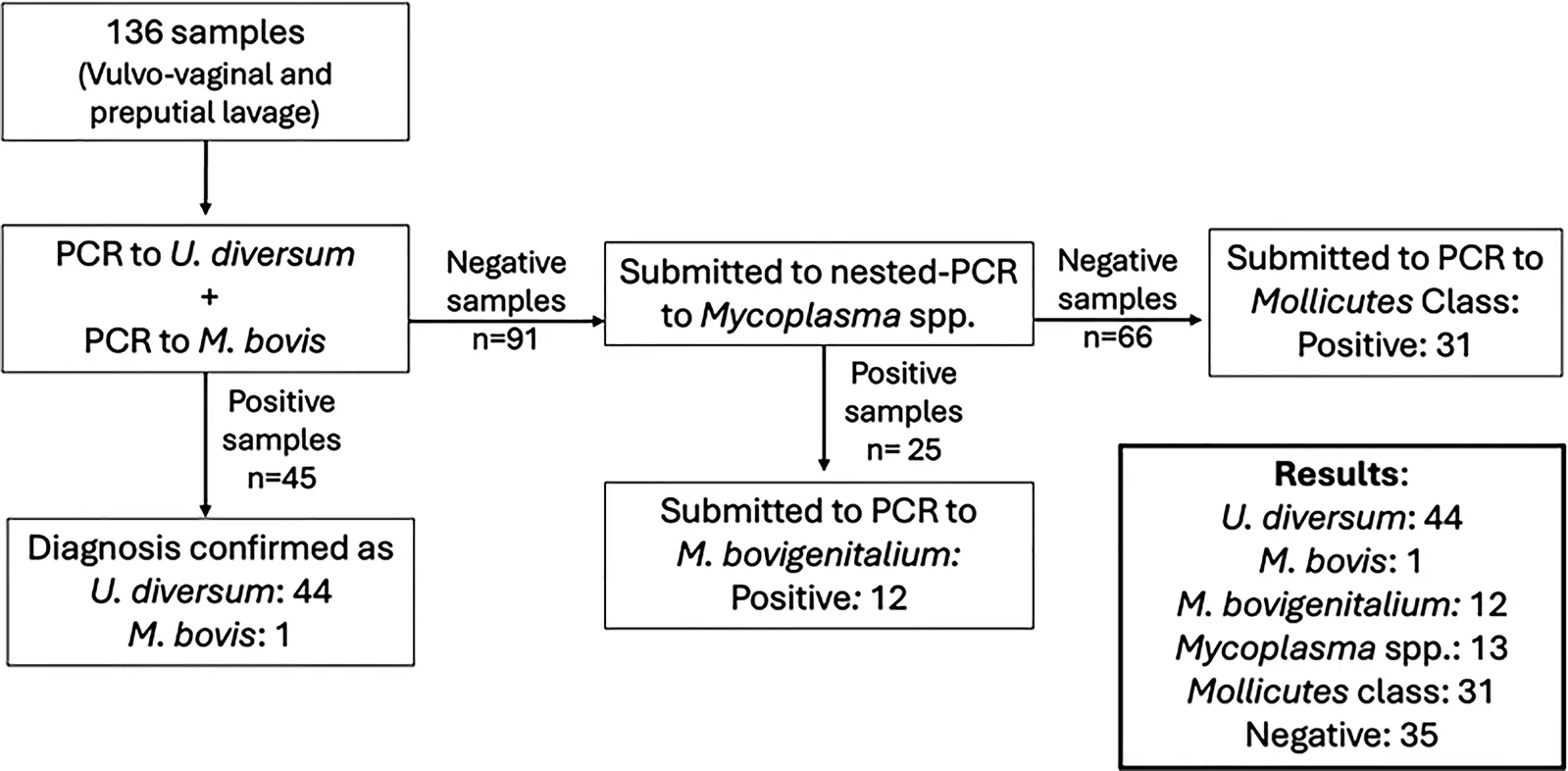
Flowchart illustrating the sequence of molecular tests performed on the 136 analyzed samples, including PCR assays for *Ureaplasma diversum*, *Mycoplasma bovis*, *Mycoplasma* spp., *Mycoplasma bovigenitalium*, and *Mollicutes*

Samples were tested in the following order: PCR for *Ureaplasma diversum* and *Mycoplasma bovis*; negative samples were subjected to PCR for *Mycoplasma* spp.; samples positive for *Mycoplasma* spp. were subjected to PCR for *Mycoplasma bovigenitalium*; samples negative for the previously tested agents were analyzed using a generic PCR for detection of the *Mollicutes* class. The testing flowchart can be viewed more clearly in Fig. 1.

**Table 1:** Primers used in PCR tests to detect *Mollicutes* class agents

Agents	Primer	DNA sequences (5’–3’)	Product (pb)
*U. diversum* ^*^	UD1	CCGGATAATAACATTTACTT	986
	UD2	CCTTGCGGTAGCAGTATCGA	
*M. bovis* ^*#*^	MB_F	GTTTGATCCTGGCTCAGGAT	198
	MB_R	CAAACGCTTCCTTTTATATTAC	
*M. bovigenitalium* ^*#*^	MBG_F	GTTTGATCCTGGCTCAGGAT	476
	MBG_R	AAGGTACATTCAATATAGTGG	
*M.* spp.^†^ (nested)	F1	ACACCATGGGAG(C/T)TGGTAAAT	350
	R1	CTTC(A/T)TCGACTT(C/T)CAGACCCAAGGCAT	
	F2	GTG(C/G)GG(A/C)TGGATCACCTCCT	
	R2	GCATCCACCA(A/T)A(A/T)AC(C/T)CTT	
*Mollicutes* ^‡^	MGSO	TGCACCATCTGTCACTCTGTTAACCTC	270
	GPO	GGGAGCAAACAGGATTAGATACCC	

^*^Cardoso et al. (2000); ^*#*^Tramuta et al. (2011); ^†^Sasaki et al. (1996); ^‡^Kuppeveld et al. (1993)

All PCR reactions were performed in a final volume of 25 µL, using 1× reaction buffer, 2.5 mM MgCl_2_, 10 mM dNTPs, 10 pmol of each primer, 2 µL of template DNA (100–200 ng), 1 U of Taq DNA polymerase, and H_2_O q.s.p. *M. bovis* and *U. diversum* samples underwent an initial denaturation at 94 °C for 1 min, followed by 35 cycles of denaturation at 94 °C for 1 min, annealing at 50 °C for 1 min, and extension at 72 °C for 1 min; and a final extension of 5 min at 72 °C.

Negative samples from the individual PCRs for *M. bovis* and *U. diversum* were tested in nested-PCR for *Mycoplasma* spp. in two cycles of an initial denaturation (94 °C for 30 s), followed by 30 cycles of 94 °C for 30 s, 55 °C for 2 min, and 72 °C for 2 min; and a final extension of 2 min at 72 °C; the second cycle was comprised of initial denaturation (94 °C for 30 s), followed by 30 cycles of 94 °C for 30 s, 56 °C for 2 min, and 72 °C for 2 min, and a final extension of 2 min at 72 °C. *Mycoplasma* spp. positive samples were tested to *M. bovigenitalium* via specific-PCR by initial denaturation at 94 °C for 1 min, followed by 35 cycles of 94 °C for 1 min, 50 °C for 1 min for primer annealing, and 72 °C for 1 min for chain extension; and a final extension of 5 min at 72 °C.

Finally, samples that remained negative in the nested PCR for *Mycoplasma* spp. were subjected to a conventional PCR for *Mollicutes* (initial denaturation at 94 °C for 5 min, followed by 35 cycles of 94 °C for 30 s, 55 °C for 30 s, and 72 °C for 30 s; final extension of 10 min at 72 °C). Positive controls included DNA from *M. bovis*, *M. bovigenitalium*, and *U. diversum* obtained from clinically confirmed samples by sequencing. Free from contamination, ultrapure water was used as a negative control.

The PCR products were visualized on a 2% agarose gel, stained with a fluorescent dye (GelRed, Biotium©, USA), and subjected to electrophoresis for 1 h at 80 V before detection under ultraviolet light on a transilluminator.

### Statistical analysis

The relationship between the detection of *Mollicutes* in the bovine reproductive tract of herds and the presence or absence of a history of reproductive disorders was analyzed using Prism 9 (version 9.5.1, GraphPad, USA), employing Fisher’s exact test. Statistical significance was set at *p* < 0.05.

## Results and discussion

**Fig. 2:**
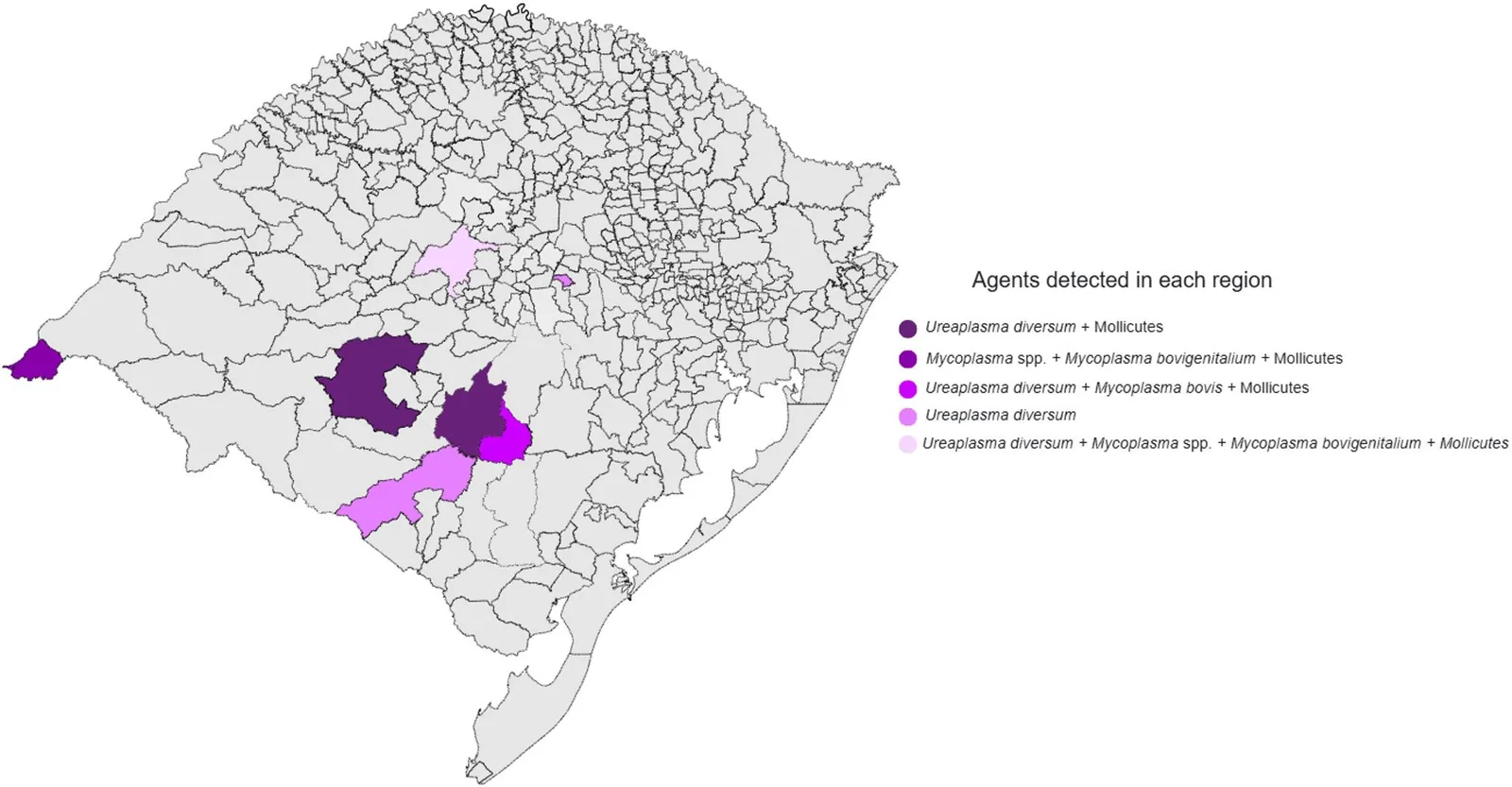
Geographic distribution of sampled farms in Rio Grande do Sul, Brazil, showing the herds positive for *Mollicutes* and associated agents (*U. diversum*, *Mycoplasma* spp., *M. bovigenitalium*, and *M. bovis*)

The analysis revealed *U. diversum* in 44 samples (32%), *M. bovigenitalium* in 12 samples (9%), and *M. bovis* in 1 sample (1%). Nested-PCR for *Mycoplasma* spp. identified 25 positive samples (18%), while 31 samples (23%) tested positive for *Mollicutes* class (Table 2). The geographic distribution of positive herds are shown in Fig. 2.

**Table 2:** Clinical and epidemiological findings of cases of *Mollicutes* in cattle farms of Rio Grande do Sul (2022–2024)

Locality	Animals	Sample	Clinical history	Tests results				
				UD	MB	MYC	MBG	MOL
Bagé	29	VS	LPM and VV	29	0	0	0	0
Barra do Quaraí	17	PL	LPM	0	0	15	3	1
Caçapava do Sul 1	05	VS	VV	1	0	0	0	4
Caçapava do Sul 2	05	VS	SM	0	0	0	0	2
Caçapava do Sul 3	05	VS	VV	0	0	0	0	4
Caçapava do Sul 4	05	VS	SM	1	0	0	0	1
Caçapava do Sul 5	05	VS	VV	1	0	0	0	0
Caçapava do Sul 6	05	VS	VV	0	0	0	0	2
Caçapava do Sul 7	05	VS	SM	2	0	0	0	4
Caçapava do Sul 8	05	VS	VV	0	0	0	0	3
Caçapava do Sul 9	05	VS	VV	0	0	0	0	5
Herveiras	05	VS	SM	1	0	0	0	0
Júlio de Castilhos	07	PL	LPM	0	0	0	7	0
Júlio de Castilhos	18	VS	LPM	5	0	10	2	1
Santana da Boa Vista	10	VS	VV	3	1	0	0	3
São Gabriel	05	VS	SM	1	0	0	0	1
Total	**136**			**44**	**1**	**25**	**12**	**31**

VS= Vaginal swab; PL= Prepucial lavage; LPR = Low pregnancy rate; VV = vulvovaginitis; SM = sanitary monitoring; UD = *U. diversum*; MB = *M. bovis*; MYC = *Mycoplasma* spp.; MBG = *M. bovigenitalium*; MOL = *Mollicutes*

All herds tested positive for at least one agent, with 56% (9/16) testing positive for *U. diversum*, 25% (4/16) for *M. bovis*, *M*. *bovigenitalium*, or *Mycoplasma* spp., and 75% (12/16) for *Mollicutes*. The latter likely do not classify as *U. diversum*, *M. bovis*, *M*. *bovigenitalium*, or another *Mycoplasma* spp., as *Mollicutes* positive samples tested negative to other agents.

The analysis of preputial lavage from bulls at the Barra do Quaraí farm, which had a documented low pregnancy rate, revealed that 15 samples tested positive for *Mycoplasma* spp. Among these, three were confirmed by nucleotide sequencing as *Mycoplasma canadense* (1/15), *M. bovigenitalium* (1/15), or *Mycoplasma californicum* (1/15). The preputial lavage samples (7/7) and some vulvovaginal swabs (2/18) from farms in Júlio de Castilhos, submitted for laboratory analysis due to concerns about low pregnancy rates, tested positive for *M. bovigenitalium*. Several species of *U. diversum* have been demonstrably associated with seminal vesiculitis, balanoposthitis, and epididymitis (Pilaszek & Truszcynski, 1988). Additionally, *M. bovigenitalium* and *M. bovis* have been shown to interfere with sperm motility and cause infertility (Cardoso & Vasconcelos, 2004), compromising the herd’s productive performance.

*U. diversum* was detected in cows with vulvovaginitis and asymptomatic animals. However, at the Bagé farm, where cows exhibited vulvovaginitis and suffered low pregnancy rates following artificial insemination, *U. diversum* was detected in all sampled animals. Laboratory tests for other reproductive diseases (other *Mollicutes*, campylobacteriosis, leptospirosis, neosporosis, *Pestivirus bovis*, and *Varicellovirus bovinealpha1*) tested negative (data not shown), suggesting a potential role of *U. diversum* in the reproductive failures observed in the herd. *Ureaplasma* spp. exert pathogenic effects primarily through ammonia synthesis (Miller et al. 1994), inhibiting ciliary activity and causing oviduct and endometrial cell destruction (Thornber 1982). These conditions can manifest with or without clinical signs and include symptoms such as granular vulvitis, endometritis, salpingitis, abortion, infertility, and embryo loss (Azevedo, 2017).

Although *U. diversum* is the primary *Mollicutes* agent associated with vesicular and granular lesions on the vulva of cows (Cardoso & Vasconcelos, 2004), it was not detected on four farms where cows exhibited these lesions (Table 2). Nevertheless, these cows tested positive for the *Mollicutes* class by PCR, suggesting an involvement of other species. *M. canadense* has been identified in cows with granulopustular lesions on the vaginal mucosa (Lysnyansky et al. 2009). Additionally, these species may have contributed to the herd’s clinical condition.

Coinfections were detected in seven herds, typically involving *U. diversum* and another agent from the *Mollicutes* class. Multiple agents are common in infectious diseases (Alfieri and Alfieri 2017). Thus, *Mollicutes* coinfections in the samples are not surprising, given that some of these microorganisms are part of the vulvar and vaginal microbiota and can exhibit opportunistic pathogenicity (Junior et al. 2021). These microorganisms can persist in a commensal state until factors such as immunosuppression, antibiotic therapy, or microbiota alterations create favorable conditions for their proliferation (Cardoso and Vasconcellos 2004). Carli et al. (2021) suggest that *M. bovis* can facilitate colonization by *U. diversum*, a phenomenon that may have occurred in this study, as both pathogens were found in coinfections.

Regarding *Mycoplasma* spp., our findings align with those observed by Buzinhani, Timenestsky, and Metiffogo (2007), who reported a 12.5% prevalence in herds in Brazil. Nevertheless, the detection rate of *U. diversum* (32%) exceeded that reported by Carli et al. (2021) in preputial lavage samples from bulls (28%) across Brazil and by Argue et al. (2013), in Australian cattle (15%).

The results indicate the widespread distribution of *Mollicutes* class in the reproductive tracts of cows and bulls in southern Brazil. Although these agents are common in cattle worldwide (Tamiozzo et al. 2014; Szacawa et al. 2018; Deeney, 2021), reports of these infections in Brazil remain scarce, and few cases have been confirmed in Rio Grande do Sul (Carli et al. 2021). Due to underreporting, these agents are often overlooked in diagnostic considerations and bovine reproductive disease control programs. Disease control strategies could include sanitary protocols, such as using double insemination pipettes and/or condoms during artificial insemination (Cardoso & Vasconcelos, 2004).

**Fig. 3:**
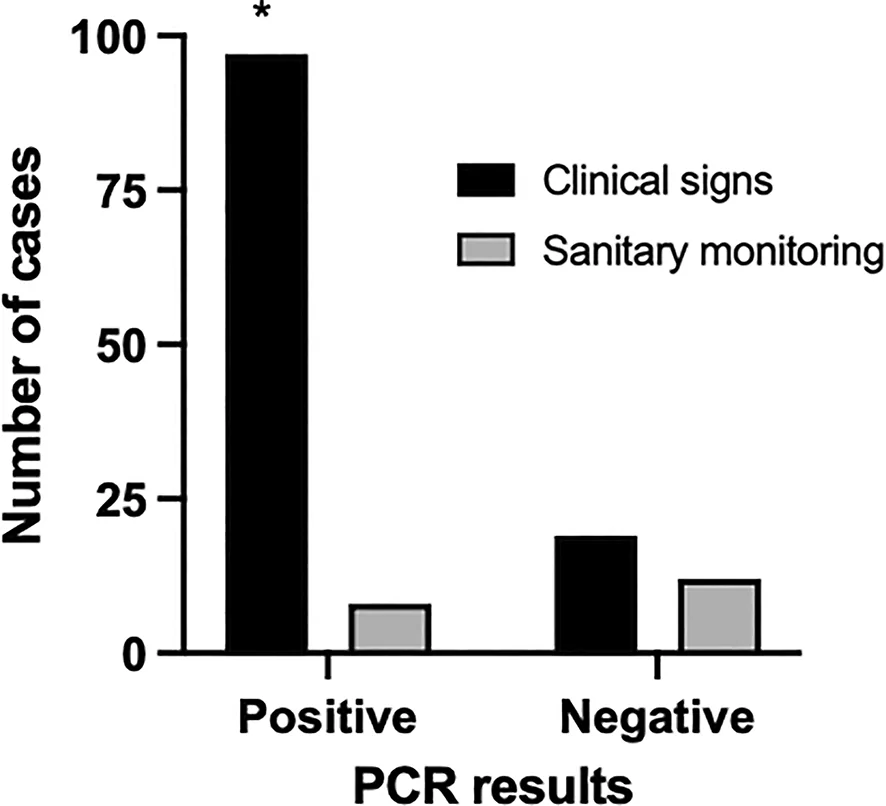
PCR results and clinical status for *U. diversum*, *M. bovigenitalium*, *M. bovis*, *Mycoplasma* spp., and/or *Mollicutes*. ^*^Association between the variables (Fisher’s exact test, *p* < 0.05). Definition: Black and gray graphic with no shading. Graphic program: Graphpad prism

Many symptomatic animals tested positive, with PCR results linking *Mollicutes* detection to clinical signs, suggesting symptomatic animals are more likely to test positive (Fig. 3). Furthermore, the results underscore the importance of asymptomatic positive animals, which can be an infection source in other animals. Among the sampled farms, 6% had animals that tested positive for *Mollicutes* despite showing no clinical signs. Due to their asymptomatic condition, these animals are often used in reproductive season and may transmit the agents. Moreover, because asymptomatic animals lack clinical signs, they are unlikely to undergo routine laboratory testing, contributing to the underdiagnosis of these infections.

Although control measures and therapeutic approaches for *Mollicutes*-infected animals remain the same regardless of the species identified in the genital mucosa (Cardoso & Vasconcelos, 2004), identifying the etiological agent is essential for understanding the epidemiology and distribution of these pathogens within herds. This knowledge aids in developing species-specific diagnostic methods and vaccines for infections caused by particular *Mollicutes* species.

Lastly, our findings confirm the distribution of different *Mollicutes* species in genital infections of cattle in Rio Grande do Sul. One potential limitation is the reliance on samples submitted to our laboratory for diagnosis rather than those collected exclusively for this experiment, which may introduce geographic bias. However, the analyzed area is representative of beef cattle farming in the state, as all significant cattle-producing regions share characteristics similar to those of the sample collection sites. Another limitation is the lack of systematic sanitary monitoring of cows with and without lesions from the time of infection diagnosis, often coinciding with the day of artificial insemination, throughout the end of the reproductive period. Therefore, it is not possible to conclude that any observed reproductive impairments were caused by the agents present in the vaginal mucosa.

## Conclusion

The results demonstrated the distribution of different species of *Mollicutes*, especially *U. diversum*, in the reproductive system of cattle across central, western, and southern Rio Grande do Sul (southern Brazil), regardless of clinical signs. Although a higher frequency of positive results was observed in herds with reproductive disorders, molecular detection alone is insufficient to establish a definitive association between the presence of the agent and the development of clinical signs.

## Data Availability

All data generated or analysed during this study are included in this published article.
